# Supplementary material for: Whole-Genome Sequence Analysis of Candida glabrata Isolates from a Patient with Persistent Fungemia and Determination of the Molecular Mechanisms of Multidrug Resistance
Source: J Fungi (Basel). 2023 Apr 26;9(5):515. doi: 10.3390/jof9050515 (PMC10218836; doi:10.3390/jof9050515)
Supplement: Supplementary file 1 [file jof-09-00515-s001.zip › jof-2347896-supplementary.pdf]

Supplementary Table S1. The list of possible genes associated with antifungal resistance in *C. glabrata*

Possible antifungal resistance genes										
ADA2	*CAGL0B03355g*	*CAGL0G07689g*	*CAGL0K03377g*	*CAGL0L10318g*	*EPA3*	*FCY2*	*INO1*	*PDR13*	*SSD1*	*YOR1*
ADH1	*CAGL0B03421g*	*CAGL0G09273g*	*CAGL0K04301g*	*CAGL0L10604g*	*ERG1*	*FEN1*	*IPC1*	*PFK1*	*STB5*	*YPS5*
ADK1	*CAGL0C02981g*	*CAGL0G09603g*	*CAGL0K05995g*	*CAGL0L10736g*	*ERG2*	*FKS1*	*JJJ1*	*PGK1*	*STR3*	
AHP1	*CAGL0D00946g*	*CAGL0H02387g*	*CAGL0K08866g*	*CAGL0M01870g*	*ERG3*	*FKS2*	*MEC3*	*PUP1*	*SUR2*	
AMT1	*CAGL0D06512g*	*CAGL0H05181g*	*CAGL0K09460g*	*CAGL0M04631g*	*ERG4*	*FKS3*	*MET8*	*PYC1*	*SUR4*	
AP1	*CAGL0E00803g*	*CAGL0H06809g*	*CAGL0K09702g*	*CAGL0M05005g*	*ERG5*	*FLR1*	*MGE1*	*QDR2*	*SUT1*	
AQR1	*CAGL0E01353g*	*CAGL0H08866g*	*CAGL0K10934g*	*CAGL0M07766g*	*ERG6*	*FLR2*	*MSH2*	*ROX1*	*TDH3*	
ATF2	*CAGL0E04554g*	*CAGL0I01980g*	*CAGL0K11616g*	*CAGL0M08426g*	*ERG7*	*FPS1*	*MT-II*	*RPN4*	*TOG1*	
BMT2	*CAGL0E04576g*	*CAGL0I02464g*	*CAGL0K12100g*	*CAGL0M09713g*	*ERG8*	*FPS2*	*NCE103*	*RSB1*	*TPO1_1*	
BRE5	*CAGL0F04917g*	*CAGL0I04422g*	*CAGL0K12958g*	*CAGL0M10219g*	*ERG9*	*GAS1*	*NDT80*	*RTA1*	*TPO1_2*	
CAGL0A01650g	*CAGL0F05467g*	*CAGL0I07249g*	*CAGL0L01485g*	*CAGL0M12749g*	*ERG10*	*GLN3*	*NOP8*	*RTT106*	*TPO3*	
CAGL0A02816g	*CAGL0F06897g*	*CAGL0I07645g*	*CAGL0L02211g*	*CAGL0M12925g*	*ERG11*	*GPD2*	*NUD1*	*SDH2*	*TPO4*	
CAGL0A04169g	*CAGL0F07117g*	*CAGL0I10604g*	*CAGL0L03135g*	*CAGL0M14047g*	*ERG13*	*HAL9*	*OCH1*	*SEC53*	*UFD1*	
CAGL0A04543g	*CAGL0G01122g*	*CAGL0J00363g*	*CAGL0L03223g*	*CAGL0M14091g*	*ERG20*	*HFD1*	*PCK1*	*SET1*	*UGP1*	
CAGL0A04829g	*CAGL0G03861g*	*CAGL0J00451g*	*CAGL0L06864g*	*CDR1*	*ERG25*	*HSC82*	*PDC*	*SHM2*	*UPC2A*	
CAGL0B01078g	*CAGL0G05269g*	*CAGL0J00891g*	*CAGL0L07678g*	*CTA1*	*ERG26*	*HSP12*	*PDH1*	*SLG1*	*UPC2B*	
CAGL0B01969g	*CAGL0G06468g*	*CAGL0J07502g*	*CAGL0L08338g*	*ECM4*	*ERG27*	*HSP31*	*PDR1*	*SNQ2*	*VPH2*	
CAGL0B02343g	*CAGL0G07271g*	*CAGL0K02563g*	*CAGL0L10186g*	*ENO1*	*FCY1*	*IFA38*	*PDR12*	*SPE3*	*YBT1*	

Supplementary Table S2. Sequencing and post-sequencing parameters of whole-genome sequencing in this study

	Sequencing parameter				Post-sequencing parameter				
Isolate no.	Total yield (Gb)	Cluster PF (%)	Cluster count PF (million)	≥Q30 (%)	Total reads	Trimming rate (%)	Mapping rate (%)	Duplicate rate (%)	Coverage
1	8.42	90.29 ± 0.73	26.81	93.57	5,172,324	0.07	98.2	0.05	60.6X
2					5,265,030	0.05	98.5	0.04	62.1X
3					5,979,756	0.06	98.7	0.04	70.8X
4					5,349,312	0.05	98.3	0.05	62.8X
5					4,772,064	0.06	98.6	0.03	56.4X
6					5,394,634	0.05	98.6	0.04	63.8X
7					4,684,438	0.05	98.6	0.04	55.4X
8					5,841,744	0.05	98.7	0.04	69.1X
9					4,739,146	0.04	98.7	0.04	56.1X
10					5,026,660	0.06	98.6	0.04	59.3X
Average	8.42	90.29 ± 0.73	26.81	93.57	5,222,511	0.05	98.6	0.04	61.6X

Abbreviations: PF, passing filter.

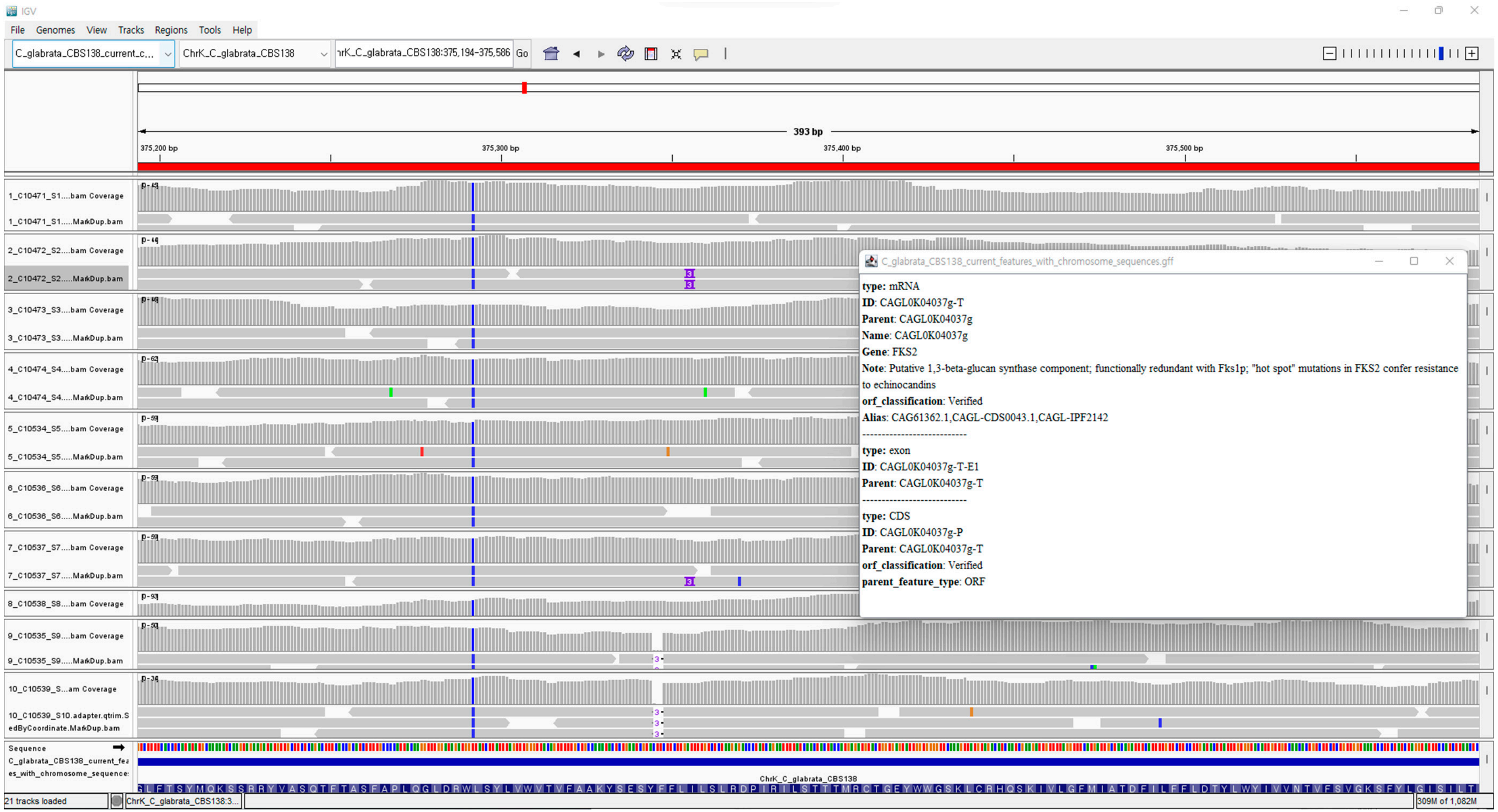

Supplementary Figure S1. An example of Integrative Genomics Viewer showing mutations on *FKS2* for 10 serial isolates of *C. glabrata.*

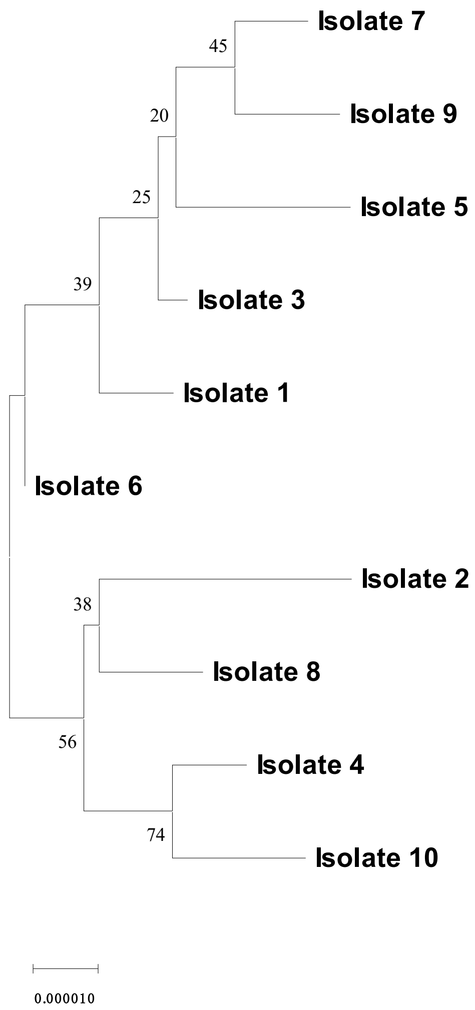

Supplementary Figure S2. Phylogenetic analysis of 10 serial *C. glabrata* isolates using SNP data for 182 resistance genes. Scale bar, number of nucleotide substitutions per site; numbers on nodes, bootstrap resampling values.
